# Neurobiological markers of early stressful events in psychosis

**DOI:** 10.1192/j.eurpsy.2024.49

**Published:** 2024-08-27

**Authors:** M. Aas

**Affiliations:** Social, Genetic and Developmental Psychiatry Centre, Institute of Psychiatry, Psychology and Neuroscience, King’s College London, London, United Kingdom

## Abstract

**Abstract:**

New data from the MRC funded project “Integrating psychological models with biological pathways in psychosis” will be presented. The overall objective of this project is to use both environmental and genetic data to understand the biological pathways in patients with schizophrenia and bipolar disorders. Specifically, to find out if polygenic risk and childhood adverse events increase the relative risk of mental illness above that of its individual case-control explained variance, and secondly, the effect of both polygenic risk and childhood adverse events on clinical characteristics and ageing processes. Both data from new unpublished systematic reviews and original data will be presented.

**Disclosure of Interest:**

None Declared

